# Supplementary material for: Polymethyl(1–Butyric acidyl)silane–Assisted Dispersion and Density Gradient Ultracentrifugation Separation of Single–Walled Carbon Nanotubes
Source: Nanomaterials (Basel). 2022 Jun 17;12(12):2094. doi: 10.3390/nano12122094 (PMC9227055; doi:10.3390/nano12122094)
Supplement: Supplementary file 1 [file nanomaterials-12-02094-s001.zip › nanomaterials-1767535-supplementary.pdf]

nanomaterials

Supporting Information for:

Polymethyl(1-Butyric acidyl)silane-Assisted Dispersion and Density Gradient Ultracentrifugation Separation of Single-Walled Carbon Nanotubes

Hongming Liu [1], Qin Zhou [1,*] and Yongfu Lian [1,*]

[1] Key Laboratory of Functional Inorganic Material Chemistry, Ministry of Education, School of Chemistry and Materials Science, Heilongjiang University, Harbin 150080, China, 2191007@s.hlju.edu.cn
* Correspondence: zhouqin@hlju.edu.cn (Q.Z.); chyflian@hlju.edu.cn (Y.L.)

1. Figures

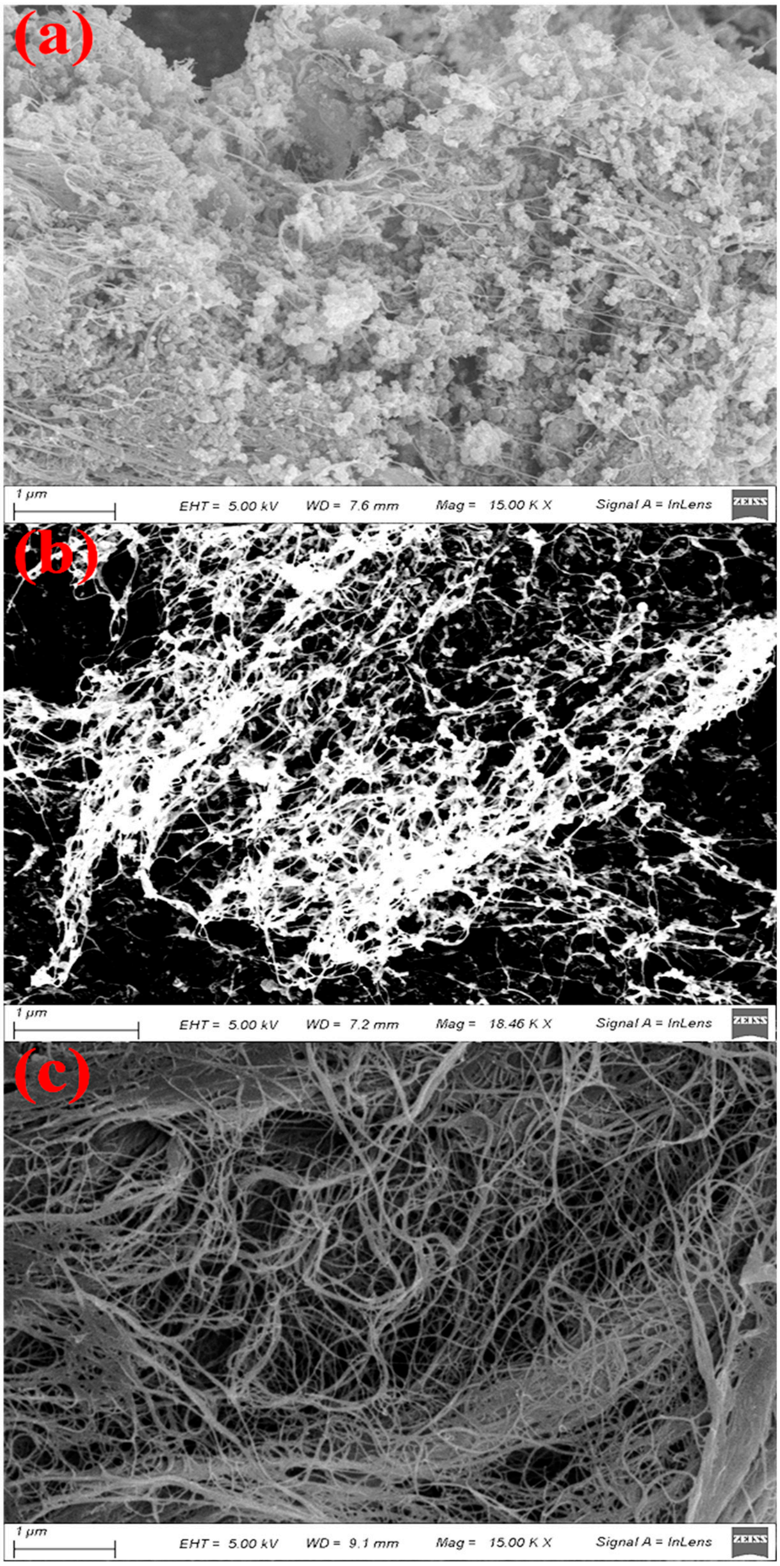

Figure S1. SEM images of (**a**) as-prepared SWNTs, (**b**) heat-treated SWNTs, and (**c**) primarily purified SWNTs, respectively.

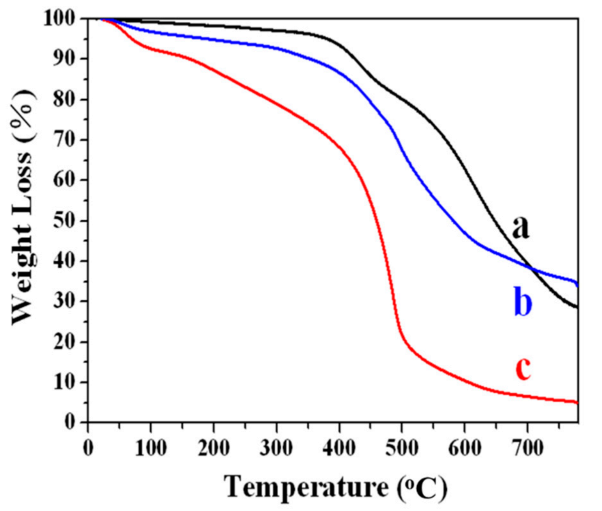

Figure S2. TG analyis of (**a**) as-prepared SWNTs, (**b**) heat-treated SWNTs, and (**c**) primarily purified SWNTs, respectively.

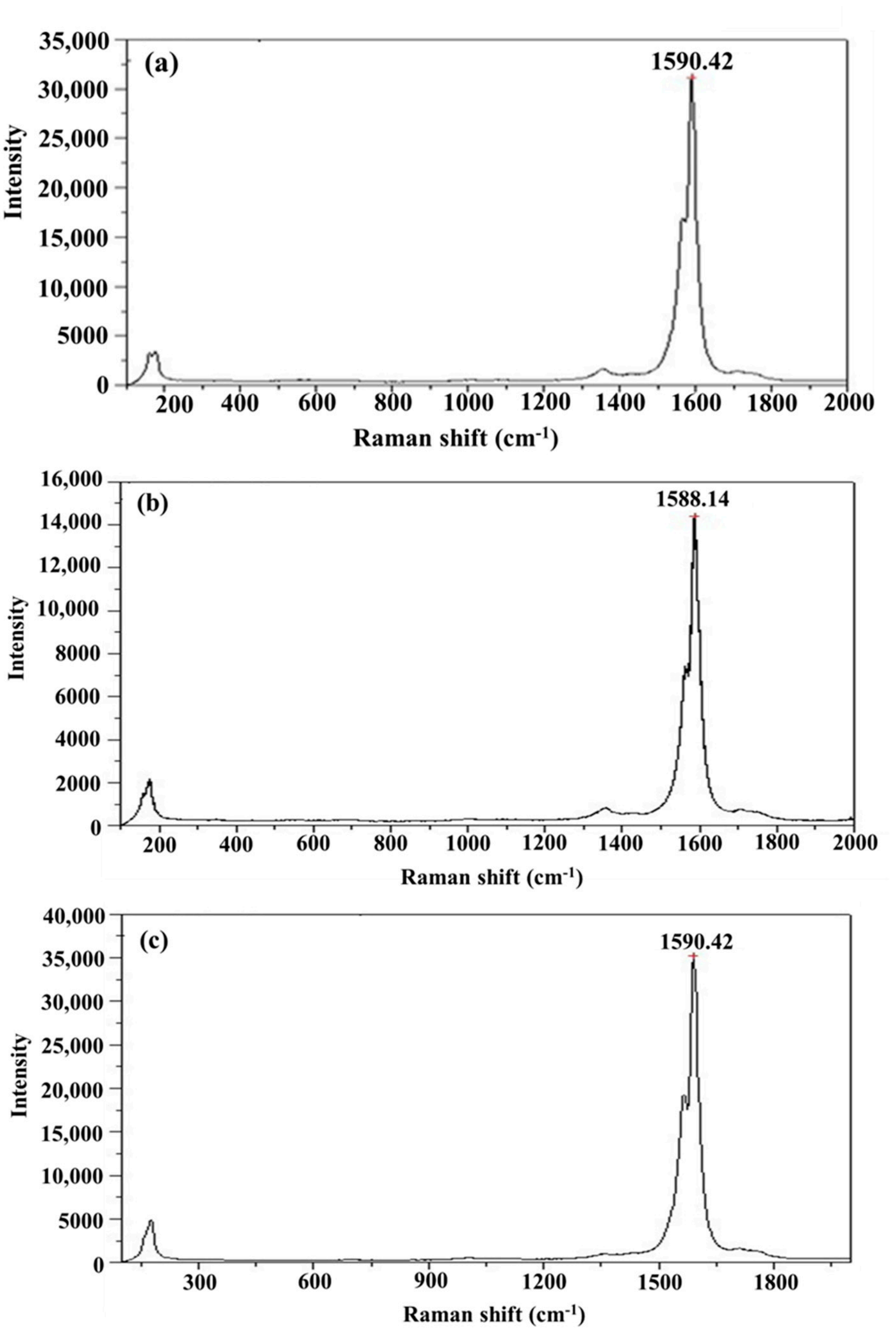

Figure S3. Raman spectra of (**a**) as-prepared SWNTs, (**b**) heat-treated SWNTs, and (**c**) primarily purified SWNTs, respectively.

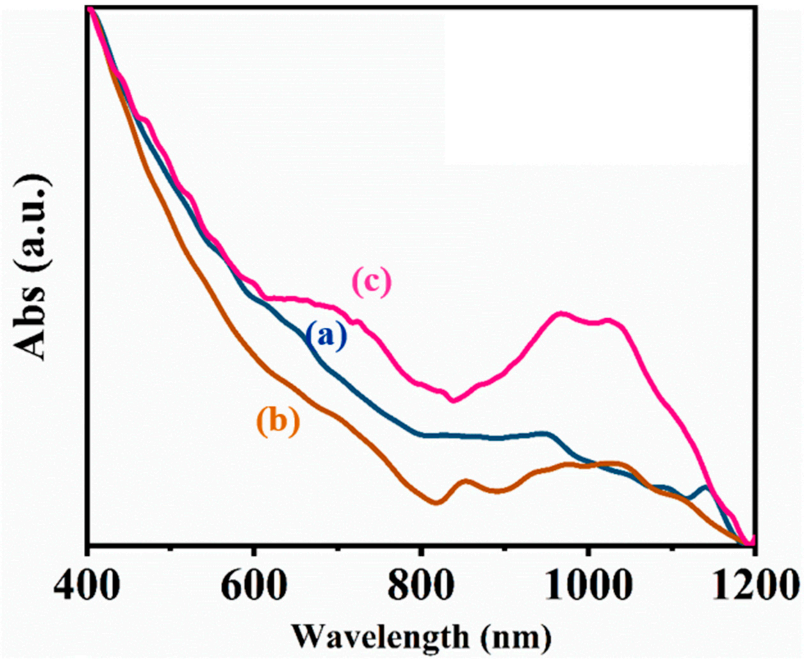

Figure S4. Vis-NIR spectra of (a) as-prepared SWNTs, (b) heat-treated SWNTs, and (c) primarily purified SWNTs, respectively.

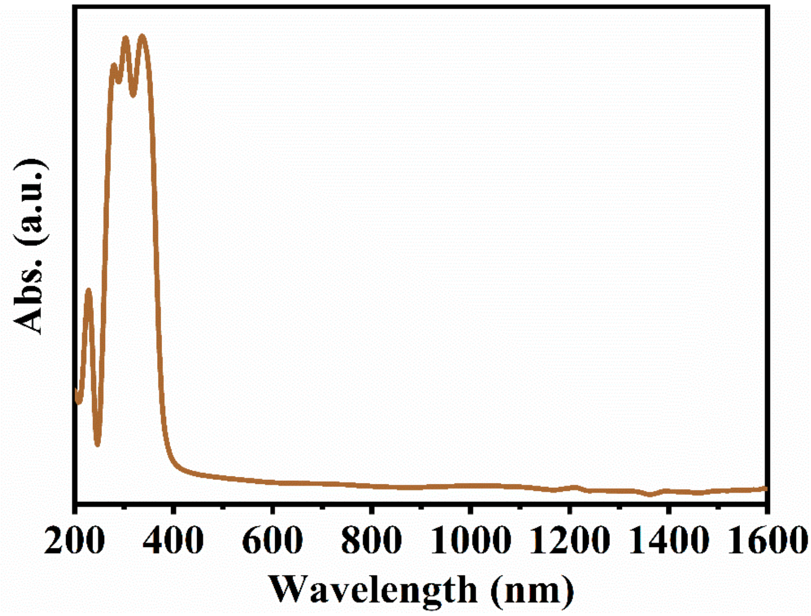

Figure S5. UV-Vis-NIR spectrum of water-soluble BA-PMS.

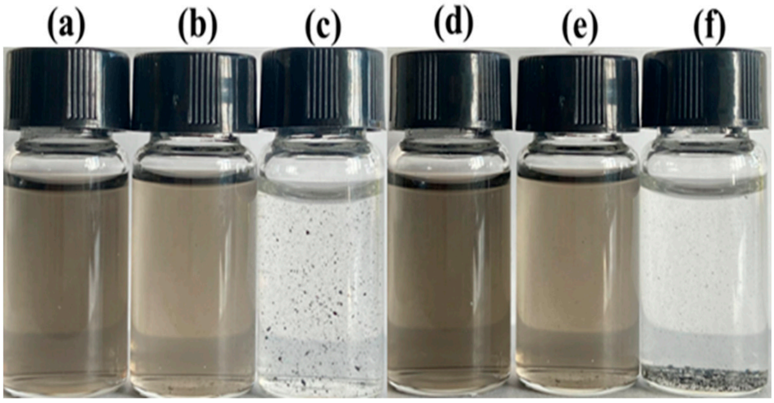

Figure S6. photographs of (**a**) SWNTs dispersed BA-PMS, (**b**) the aqueous solution of DOC, and (**c**) SWNTs dispersed in water. (**d**), (**e**) and (**f**) are taken after (**a**), (**b**) and (**c**) setting for 90 days, respectively.

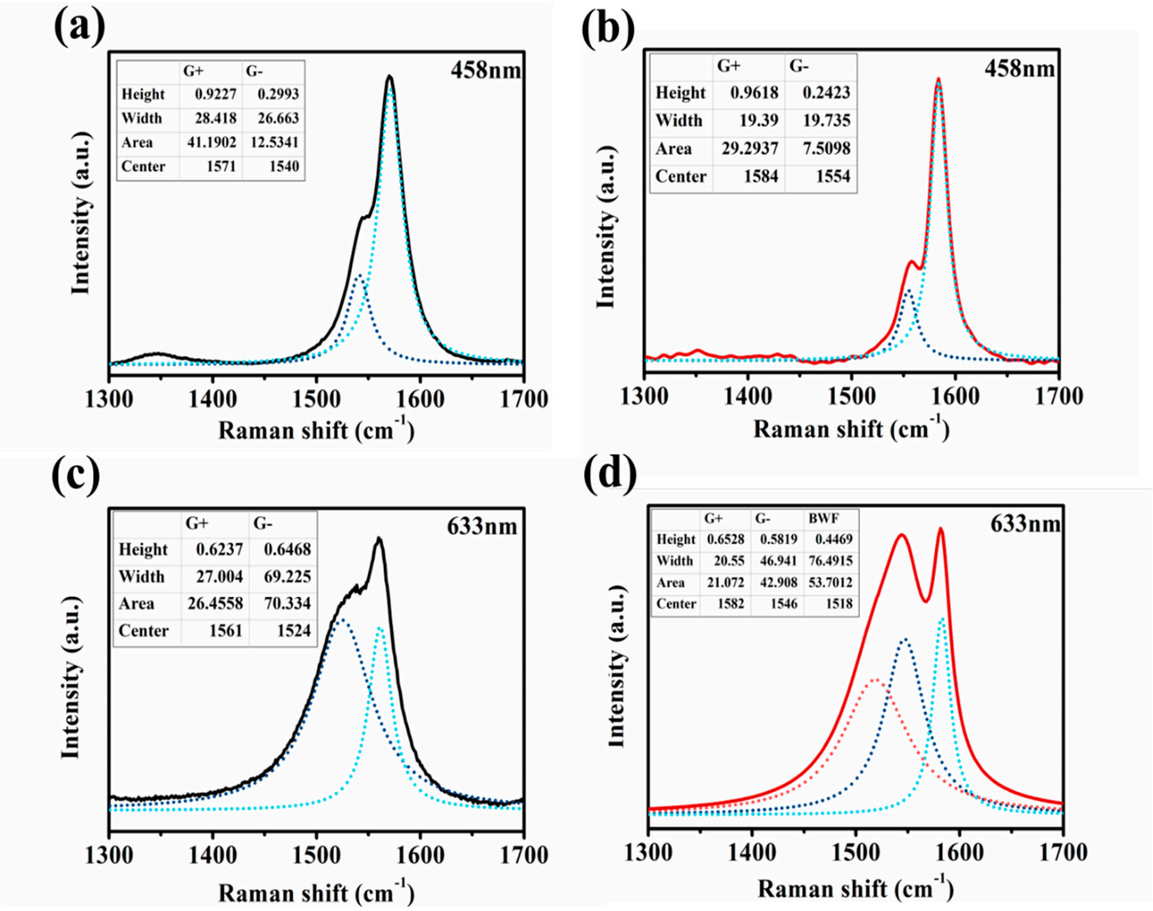

Figure S7. Lorentz-fitted curves of G-bands of primarily purified SWNTs (black lines) and BA-PMS-dispersed SWNTs (red lines) at (**a**,**b**) 458 and (**c**,**d**) 633 nm excitation wavelengths, respectively.

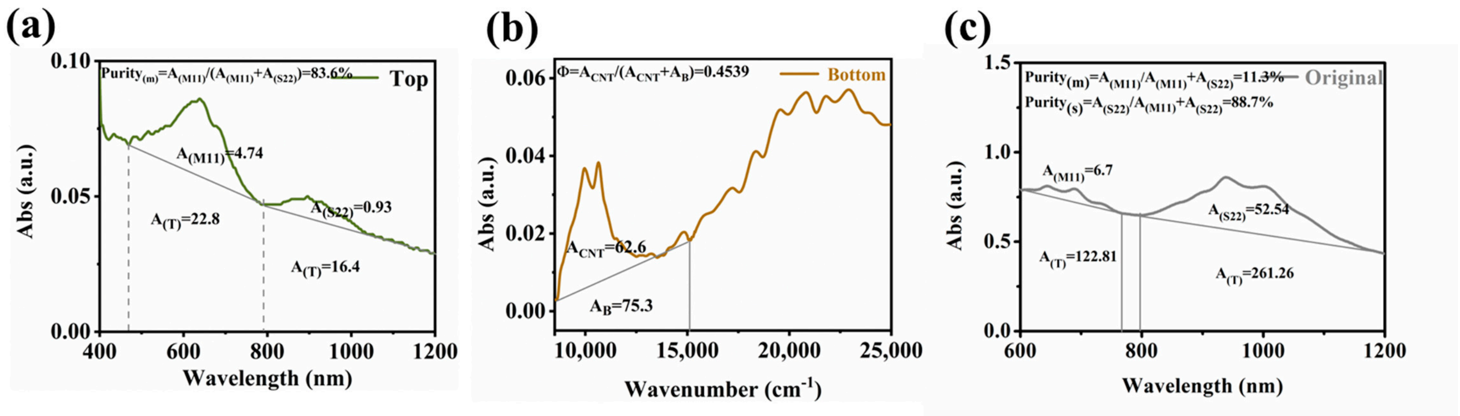

Figure S8. Purity calculation on the vis-NIR absorption spectra of (**a**) top green layer, (**b**) bottom red-brown layer, and (**c**) original SWNTs.

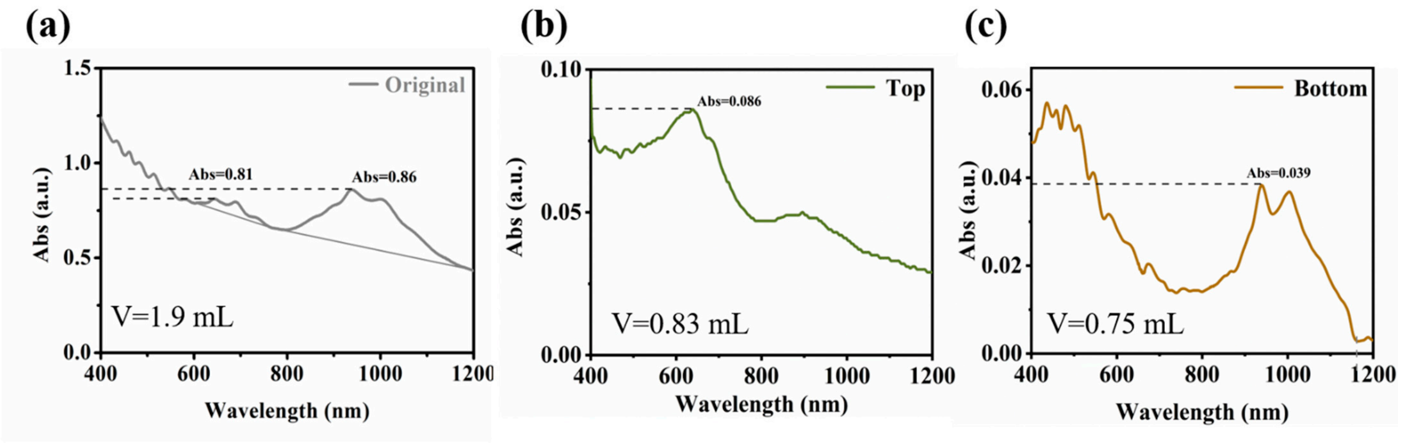

Figure S9. The absorption spectra of (**a**) SWNTs dispersion before DGU separation, DGU separated (**b**) m-SWNTs and (**c**) sc-SWNTs dispersions.

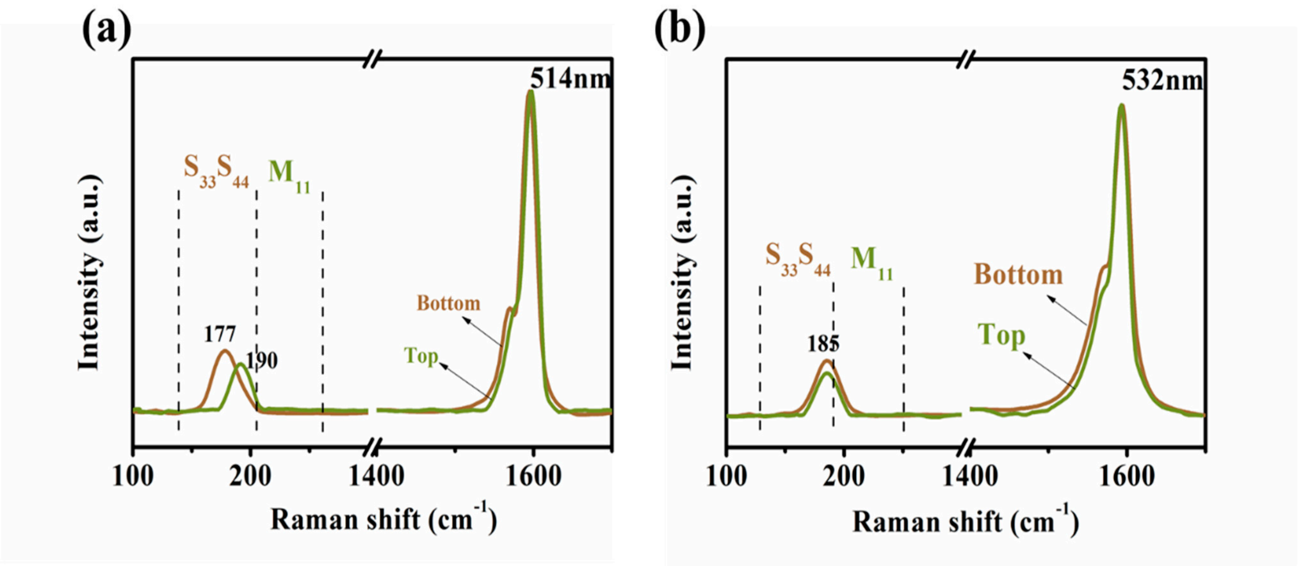

Figure S10. Raman spectra of the SWNTs in the top green layer and the bottom red-brown layer at the excitation of (**a**) 514 and (**b**) 532 nm, respectively, in the ranges of 100-1700 cm^{-1}.

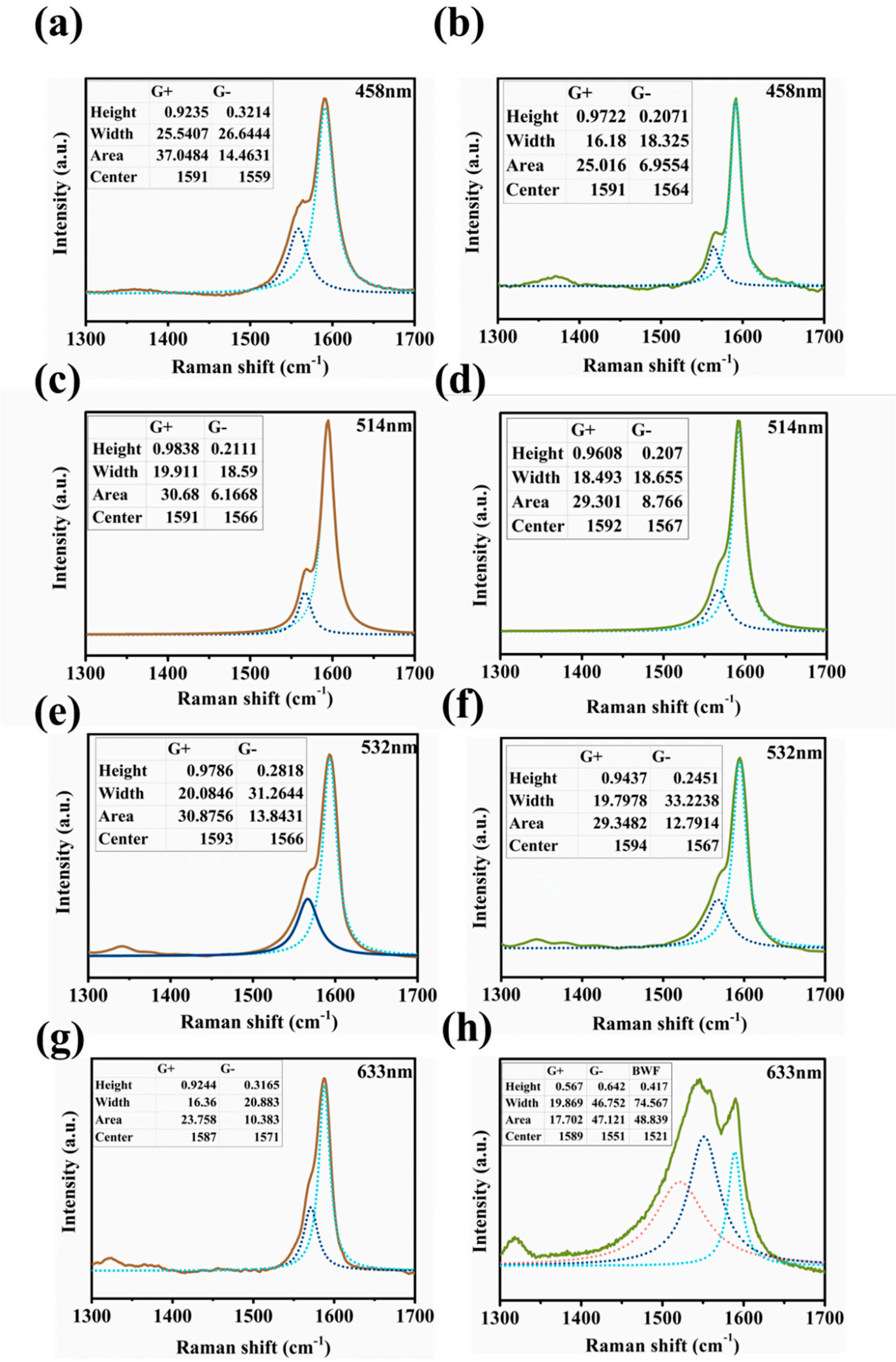

Figure S11. Lorentz-fitted curves of G-bands of the bottom red-brown layer (brown lines) and top green layer (green lines) at (a-b) 458, (c-d) 514, (e-f) 532, and (h-g) 633 nm excitation wavelengths, respectively.

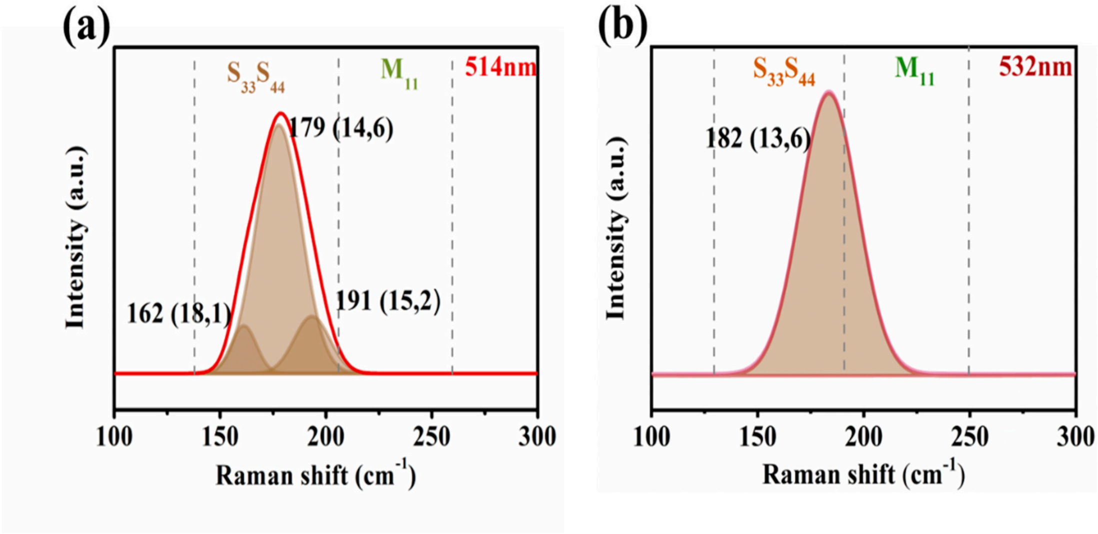

Figure S12. Raman spectra of the BA-PMS-dispersed-SWNTs at the excitations of (a) 514 and (b) 532 nm, respectively, in the range of 100-300 cm^{-1}.

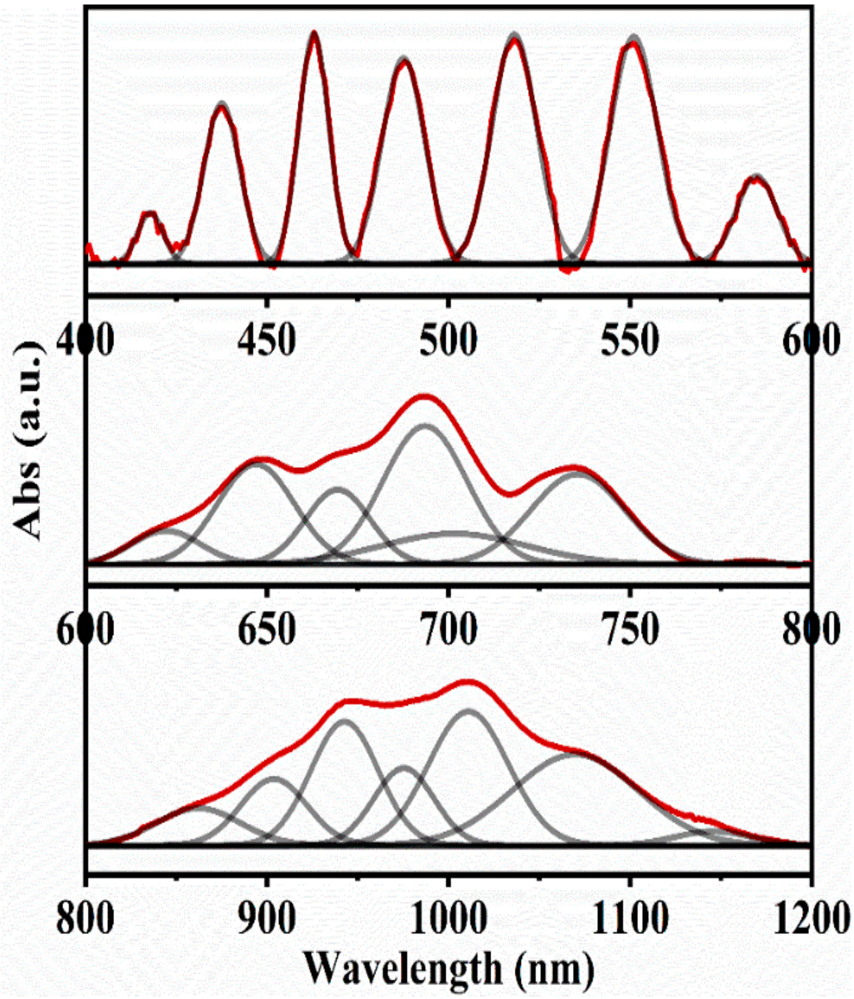

Figure S13. Gaussian fit curves of Vis-NIR absorption spectra of BA-PMS-dispersed SWNTs.

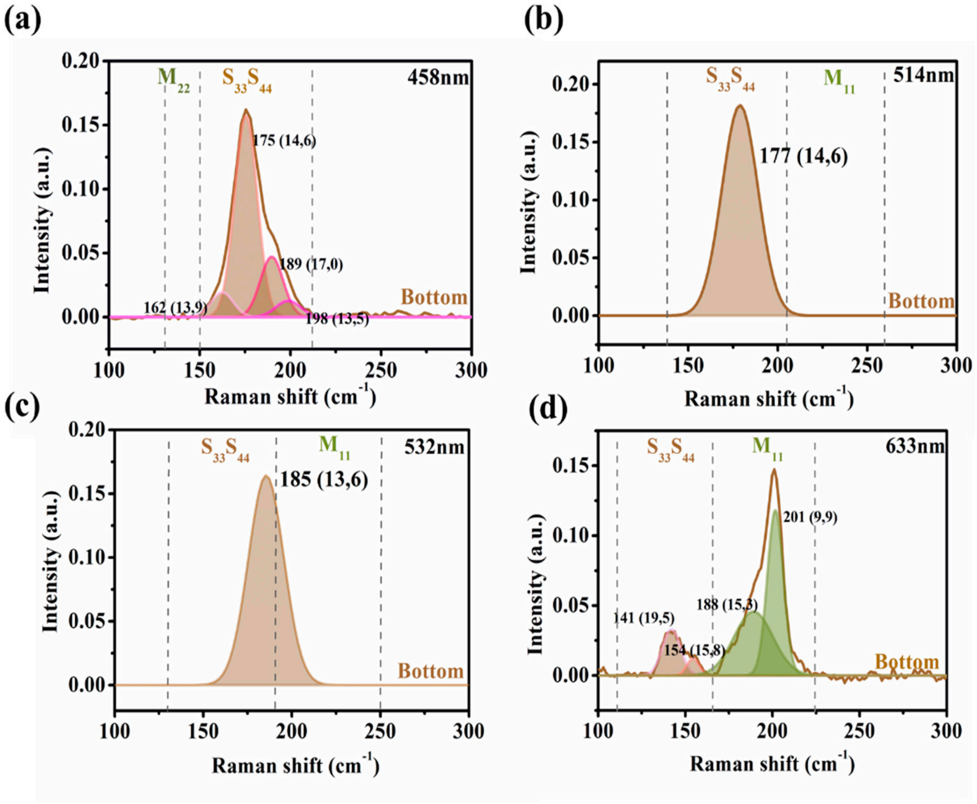

Figure S14. Gaussian fitting curves for the bottom red-brown layer at (a) 458, (b) 514, (c) 532, and (d) 633 nm excitation wavelength in the range of 100-300 cm^{-1}, respectively.

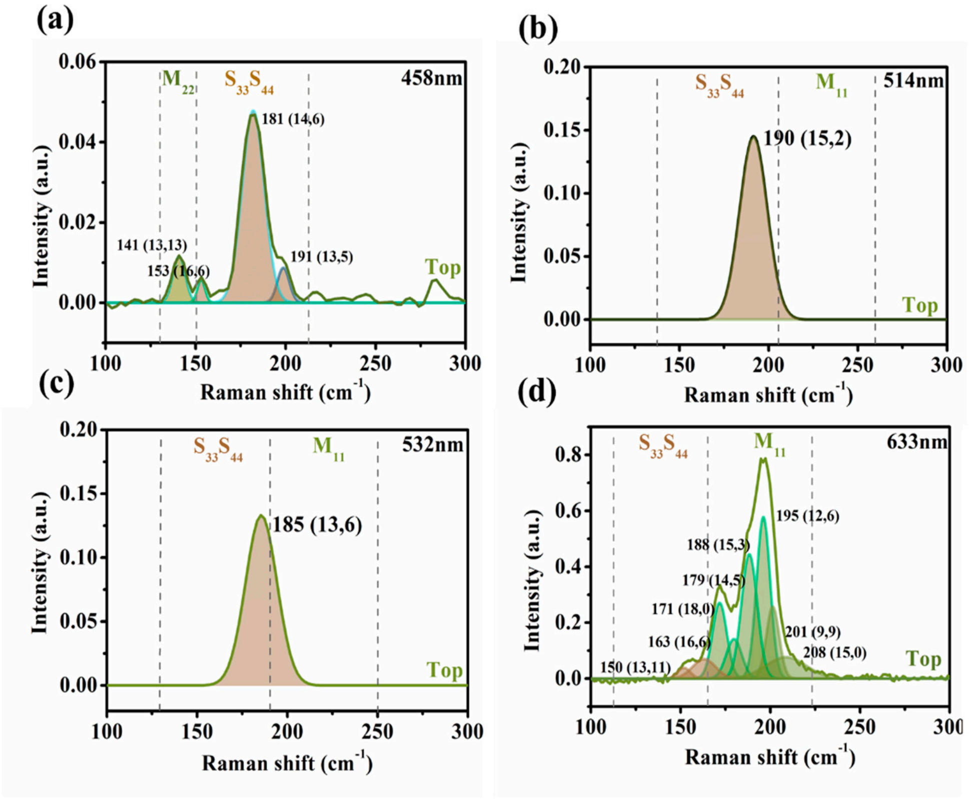

Figure S15. Gaussian fitting curves for the top green layer at (a) 458, (b) 514, (c) 532, and (d) 633 nm excitation wavelength in the range of 100-300 cm^{-1}, respectively.

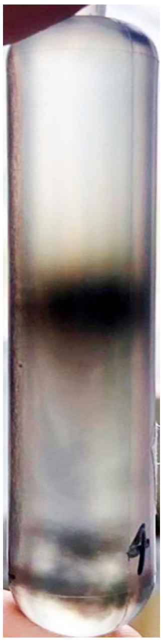

Figure S16. DGU separation of the co-surfactants (Ua-PMS and SDS) dispersed SWNTs.

2. Tables

Table S1. The performance features of DGU separation of SWNTs

Separation type	Medium	Surfactant	Enrichment	Ref.
m/sc	Iodixanol	SC, DOC, and STDOC	97% sc-SWNTs	[51]
m/sc, d	Iodixanol	SC, SDS	99% pure semiconducting CNTs with narrow diameter distributions	[60]
m/sc, (n,m)	Iodixanol	SC, SDS	Enantiomers of different SWNTs chiralities	[61]
m/sc	Sucrose	SC	95% sc-SWNTs, 69% m-SWNTs	[62]
(n,m)	Iodixanol	N-CS, SDS	SWNTs of different chirality	[46]
m/sc	Iodixanol	Pluronic, Tetronic	99% sc-SWNTs, 74% m-SWNTs	[52]
m/sc	Iodixanol	Pluronic	99% sc-SWNTs, 99% m-SWNTs	[63]
m/sc	Sucrose	BA-PMS, SDS	86.5% m-SWNTs, 99% sc-SWNTs	This Work

Table S2. Information on the chiral indices (n,m) designation of the absorption peaks in Figure S8.

Absorption peaks (nm)	Interband transitions	Calculated diameters (nm)	Chiral indices (n,m)
418	S33	1.129	(12,4)
859	S22		(12,4)
437	S33	1.169	(11,6)
902	S22		(11,6)
621	M11	1.22	(9,9)
463	S33	1.223	(10,8)
942	S22	1.23	(14,3)
973	S22	1.26	(13,5)
488	S33		(13,5)
647	M11	1.308	(15,3)
1011	S22	1.331	(17,0)
668	M11	1.336	(14,5)
692	M11	1.376	(13,7)
518	S33	1.392	(14,6)
1072	S22		(14,6)
701	M11	1.409	(18,0)
737	M11	1.467	(15,6)
551	S33	1.487	(16,5)
1147	S22	1.542	(16,6)
584	S33		(16,6)

Table S3. Summary of Raman chiral assignment information of BA-PMS dispersed SWNTs and top green and bottom brown layers at 458, 514, 532, and 633 nm excitation wavelengths.

		**Before DGU** *			
458 nm	147(19,4)<br>dt=1.663	159(16,6)<br>dt=1.542	172(14,6)<br>dt=1.392	188(17,0)<br>dt=1.331	197(13,5)<br>dt=1.260
514 nm	162(18,1)<br>dt=1.450	179(14,6)<br>dt=1.392	191(15,2)<br>dt=1.260		
532 nm	182(13,6)<br>dt=1.317				
633 nm	149(15,10)<br>dt=1.706	166(16,6)<br>dt=1.542	177(14,5)<br>dt=1.336	191(15,3)<br>dt=1.308	210(14,2)<br>dt=1.182
		**After DGU (Top green layer)** *			
458 nm	141(13,13)<br>dt=1.777	153(16,6)<br>dt=1.542	181(14,6)<br>dt=1.392	191(13,5)<br>dt=1.260	
514 nm	190(15,2)<br>dt=1.260				
532 nm	185(13,6)<br>dt=1.317				
633 nm	150(13,11)<br>dt=1.629	163(16,6)<br>dt=1.542	171(18,0)<br>dt=1.409	179(14,5)<br>dt=1.336	188(15,3)<br>dt=1.308
	195(12,6)<br>dt=1.243	201(9,9) 1.220	208(15,0)<br>dt=1.174		
		After DGU (**Bottom brown layer**) *			
458 nm	162(13,9)<br>dt=1.500	175(14,6)<br>dt=1.392	189(17,0)<br>dt=1.331	198(13,5)<br>dt=1.260	
514 nm	177(14,6)<br>dt=1.392				
532 nm	185(13,6)<br>dt=1.317				
633 nm	141(19,5)<br>dt=1.717	154(15,8)<br>dt=1.583	188(15,3)<br>dt=1.308	201(9,9)<br>dt=1.220	

* Brown is sc-SWNTs; Green is m-SWNTs; dt (nm) is the diameter of SWNTs.

3. The Recovery Yields of m- and sc-SWNTs

In addition to this, the recovery yields of m- and sc-SWNTs could be estimated by the absorbance and volume of SWNTs dispersion before and after DGU separation. According to Beers law (A = εlc), the absorbance (A) of SWNTs is directly proportional to their concentration (c) under the same extinction coefficient (ε) and path length (l). Thus, the ratio of absorbance equals to that of concentration for m- or sc-SWNTs. Considering the volumes of SWNTs dispersion before and after DGU separation, the recovery yields of m- and sc-SWNTs could be extracted by

$$\eta_m = \frac{A_m * V_m}{A1 * V} * 100\% \quad \text{or} \quad \eta_{sc} = \frac{A_{sc} * V_{sc}}{A2 * V} * 100\%$$

where, $A1$ or $A2$ is the maximum absorbance of m- or sc-SWNTs before DGU separation. A_m and V_m (A_{sc} and V_{sc}) are the maximum absorbance and volume of the m-SWNTs (sc-SWNTs) dispersion after DGU separation. V is the volume of the SWNTs dispersion before DGU separation. Based on the data shown in Figure S9, the recovery yields of m- and sc-SWNTs were estimated to be 4.6% and 1.8%, respectively.
